# Comparative genomic analysis of clinically relevant human skin-associated fungi

**DOI:** 10.21203/rs.3.rs-6700810/v1

**Published:** 2025-06-11

**Authors:** Sofie Agerbæk, Knud Nor Nielsen, Julie B.K. Sølberg, Ying Marlene Zhang, Zahra Akil Meften Al-Badran, Marc Stegger, Sonja Kabatnik, Matthias Mann, Rachel A. Clark, Ditte M. L. Saunte, Alberto Santos, Marianne Bengtson Løvendorf, Beatrice Dyring-Andersen

**Affiliations:** 1) Department of Dermatology, Zealand University Hospital, Roskilde, Denmark; 2) The Novo Nordisk Foundation Center for Biosustainability, Technical University of Denmark, Denmark; 3) Department of Dermatology and Allergy, Copenhagen University Hospital, Herlev and Gentofte Hospital, Denmark; 4) Department of Bioinformatics, Statens Serum Institut, Denmark; 5) Antimicrobial Resistance and Infectious Diseases Laboratory, Harry Butler Institute, Murdoch University, Australia; 6) Novo Nordisk Foundation Center for Protein Research, Faculty of Health and Medical Sciences, University of Copenhagen, Denmark; 7) Department of Dermatology, Mass General Brigham, Harvard Medical School, Boston, US; 8) Department of Clinical Medicine, Faculty of Health and Medical Sciences, University of Copenhagen, Denmark; 9) Leo Foundation Skin Immunology Research Center, Department of Immunology and Microbiology, Faculty of Health and Medical Sciences, University of Copenhagen, Denmark

**Keywords:** fungal genomics, superficial fungal infections, tinea, candida, dermatophyte, onychomycosis

## Abstract

Fungal skin infections represent a significant global health burden, affecting approximately one billion people annually. Despite their prevalence and major global health impact, the molecular mechanisms underlying pathogenicity remain largely uncharacterized. Here we present high quality genomic datasets for 51 fungal strains, representing highly prevalent and clinically relevant species associated with human skin infections. Comparative genomics reveal substantial variation in genome size and gene contents, indicating genome compaction occurred as the fungi transitioned from free-living to host-associated lifestyles. We report two non-hybrid strains of *Trichosporon ovoides*, the causative agent of white piedra. Our analysis reveals substantial differences in metabolic adaptations across skin-associated fungi, corresponding to distinct body-site and nutrient niches. Significant differences were also present in the distribution of virulence factors and adhesins, which are imperative for biofilm formation and antifungal resistance. We discuss metabolic adaptation and virulence mechanisms revealed by our data in the context of clinical presentations, highlighting shared and lineage-specific adaptations.

Together, these insights advance our knowledge of skin-associated fungi and their infection mechanisms while providing valuable resources and a foundation for future analyses to improve diagnostics and therapeutics for diverse diseases.

## Introduction

**Figure 1.**
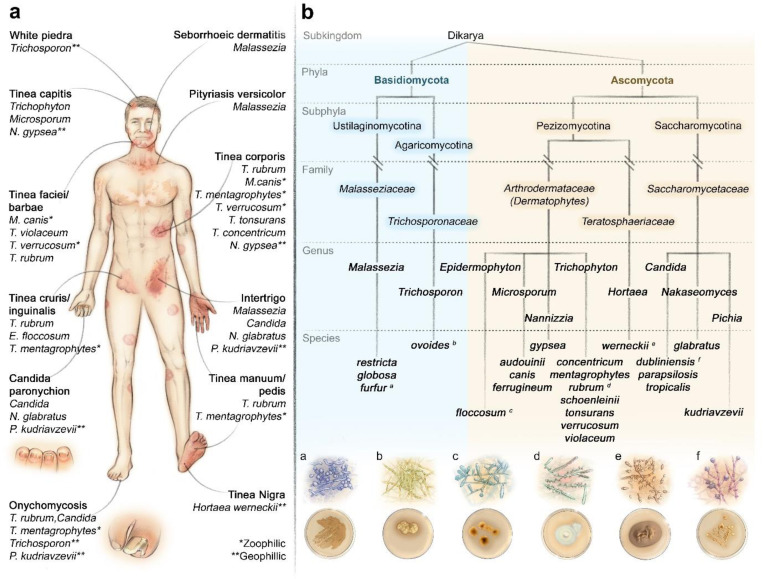
Schematic overview of clinical presentations and phylogeny of skin-associated fungal species. **(a)** Clinical presentations of fungal skin infections and their most common causative fungal group or species. Species that are not marked zoo- or geophilic with asterisks are antropophilic. **(b)** Selected taxonomic ranks of the species illustrated (taxonomic ranks written on the left). Colony appearance and microscopic morphology illustrated: *M. furfur* (a), *T. ovoides* (b), *E. floccosum* (c), *T. rubrum* (d), *H. Werneckii* (e), and *C. dubliniensis* (f).

Superficial fungal infections (SFIs) represent common dermatological conditions, impacting approximately one billion people worldwide [1]. With an estimated lifetime risk of 20%−25%, these infections consistently rank among the top ten most prevalent skin diseases and include common diseases such as ringworm and pityriasis versicolor in a broad range of niches on the human body (Figure 1). While specific fungal pathogens show distinct geographical distributions, the predominant causative organisms continue to persist worldwide. The primary etiological agents of SFIs belong to three major groups: molds of the family *Arthrodermataceae* (dermatophytes), yeasts of the family *Saccharomycetaceae*, and yeasts of the genus *Malassezia*.

Despite their significant clinical importance and worldwide distribution, several skin-associated fungi lack comprehensive genome sequencing data, and for species with available information, many reference genomes are either incomplete or unavailable in public databases. Furthermore, available genomic data frequently originates from single isolates of unknown origin, failing to capture intraspecies variation.

Diagnosis of superficial mycoses currently presents significant challenges due to their diverse yet overlapping clinical manifestations, which commonly include pruritus, pigmentation abnormalities and desquamation. Diagnostic analysis typically begins with patient history assessment and clinical evaluation, followed by direct microscopic examination of specimens (skin/nail scrapings or hair) using potassium hydroxide (KOH) preparation and time-consuming culture-based methodologies for definitive identification. When these conventional methods are inconclusive, molecular diagnostic analyses of ribosomal RNA (rRNA) or Internal Transcribed Spacer (ITS) regions of the genomes sometimes provide more precise diagnosis depending on etiology and availability. More thorough and broader genomic analyses of SFI-causing species would expand the potential for specific diagnostic tools.

Next-generation sequencing technologies have revolutionized our ability to study microbial genomes over the last decade [2]. Still, many clinically relevant, globally prevalent fungi remain poorly characterized at the genomic level. The limited availability of well-annotated genomes for skin-associated fungi has hindered comparative genomic analyses that could reveal evolutionary adaptations to the skin environment, virulence mechanisms, and the identification of novel therapeutic targets.

Despite these limitations, recent reports have revealed important differences in adaptation processes and niche specialization across species of skin-associated fungi. Different requirements for energy sources, salt tolerance, and water availability lead to distinct body-site preferences; for instance, lipophilic *Malassezia* fungi tend to inhabit sebum-rich skin regions where they cause conditions including pityriasis versicolor and seborrhoeic dermatitis [3]. Virulence mechanisms also differ across skin-associated fungi, particularly with respect to the adhesin types they express to facilitate host cell attachment and the enzymes they secrete to assimilate energy or invade tissue [4][5][6]. These differences underline the high degree of variance in host specificity and tissue tropism observed among skin-associated fungi, contributing to their diverse clinical manifestations. While genomic studies on *Candida* species have shed light on the role of adhesin diversity and virulence factors [7][8], there are limited data to support comparative genomics across skin-associated fungi, despite their high prevalence.

In this study, we present novel genome-scale data for a comprehensive collection of clinically relevant fungi associated with SFIs. The data set focuses on 51 strains of the 21 fungal species with the highest global prevalence in human skin infections. Using a comparative genomic analysis, we examine adaptation, pathogenicity and virulence by comparing the presence and copy-number variations of genes associated with these traits. By providing a comprehensive genomic resource of clinically relevant skin-associated fungi, we aim to enhance future investigations into the diagnosis and molecular mechanisms underlying SFI-associated diseases.

## Results

### Comparative genomic analysis of clinically relevant fungal species reveals non-hybrid strains of Trichosporon ovoides

To establish a genomic framework covering the most common fungi associated with SFIs in humans, we reviewed the literature to identify the most prevalent and thereby clinically relevant species worldwide (details in Methods). Our analysis identified ten predominant genera: *Candida, Epidermophyton, Hortaea, Malassezia, Microsporum, Nakaseomyces, Nannizzia, Pichia, Trichophyton,* and *Trichosporon*. Within these genera, we identified 21 species commonly associated with human superficial mycoses, representing diverse taxonomic groups and infection sites (Figure 1). To ensure clinical relevance, we targeted strains isolated from human skin or skin-related infections when available. The final cohort comprised 51 distinct fungal strains and 3 duplicates (Table 1, sheet 1) cultured and sequenced using the Illumina NextSeq 550 platform.

**Figure 2.**
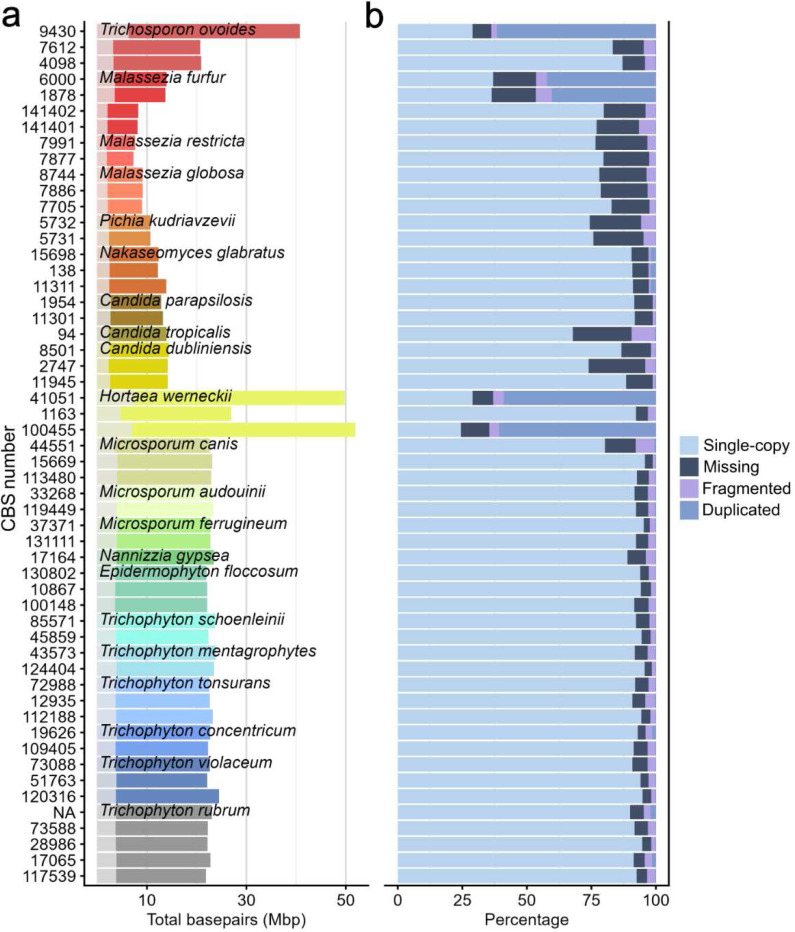
Genome size distribution and completeness assessment across skin-associated fungi. (a) Total genome sizes in megabase pairs (Mbp) for 54 genomes representing 22 species across 10 genera of skin-associated fungi. Strain identifiers (CBS numbers) are shown on the y-axis, and bars are color-coded by species. Total length of all genes for each sample is shown by the white overlay. (b) BUSCO (Benchmarking Universal Single-Copy Orthologs) analysis results showing the percentage of universal single-copy ortholog genes (light blue), multiple copies (mid blue), missing genes that may indicate gaps (dark blue) or fragmented (lilac).

The fungal genomes exhibited considerable size variation, ranging from approximately 8 Mbp in *Malassezia* species to over 50 Mbp in *Hortaea*. Among dermatophytes, we observed similar genome sizes and gene numbers, with genome sizes ranging from 21.8 Mbp in *T. rubrum* to 24.5 Mbp in one *T. tonsurans* strain. Some *H. werneckii* and *M. furfur* strains displayed a notably larger genome (~50 Mbp), consistent with previously reported genomic hybridizations [9][10] (Figure 2a).

Interestingly, two strains of *T. ovoides* (CBS7612 and CBS4098) exhibited genomes approximately half the length of a third strain (CBS9430), with the latter corresponding in size to the genomes available on NCBI genomes [11]. Previous reports argued that *T. ovoides* (and *T. coremiiforme*) are natural hybrids, with reported genomes sizes of 42 Mbp [12][13] while other members of this genus, *T. asahii* and *T. inkin* are documented non-hybrids. To validate our results, which instead support the presence of non-hybrid *T. ovoides* strains, we performed Average Nucleotide Identity analysis (OrthoANIu [14]) comparing our three isolates with reference genomes of each *ovoides* (GCA_040368805.1), *coremiiforme* (GCA_039545355.1), *asahii* (GCA_000293215.1) and *inkin* (GCA_040365635.1). Our three samples had >97.5% identity with the *ovoides* reference, and less than 85.5% identity with the three other species, confirming their classification as *T. ovoides*.

We assessed the quality and completeness of the assemblies using Benchmarking Universal Single-Copy Ortholog analysis (BUSCO [15]). The majority of genomes demonstrated high completeness scores with few missing orthologues (Figure 2b). Some cases showed missing and fragmented percentages above 20%, likely due to short-read mis-assemblies resulting from the high-repeat genomes characteristic of these fungi. Duplication percentages above 50% were observed in the hybrid genomes of *T. ovoides*, *H. werneckii* and *M. furfur*. Taken together, we assembled 54 genomes of clinically relevant skin-associated fungi, discovering previously undocumented non-hybrid strains of *T. ovoides,* the causative agent of white piedra, in the process.

### Core- and species-specific gene families in clinically relevant fungal species

To identify orthologous genes across fungal strains, we performed ortholog clustering using Orthofinder [16]. Analysis of 399,926 genes across all samples (including outgroups *Blastocladiella* and *Radiomyces)*, yielded 19,737 orthogroups (OGs), comprising 98.1% of the genes. Of these, 827 OGs were conserved across all samples while 8,528 OGs were species-specific, with 7,298 of these shared between two or more strains (Table 2). Species-specific OG distribution varied markedly from zero in two *T. violaceum* strains (CBS517.63 and CBS730.88) to 2,434 in one *H. werneckii* strain (CBS410.51).

We identified nine OGs shared across all skin-associated species (excluding the outgroups), representing conserved fungal genes across both pathogenic and non-pathogenic genera in the subkingdom Dikarya (e.g. cellulase, phospholipase, and ATP-binding protein). The absence of a common genomic signature linked to pathogenicity was expected due to the large evolutionary distance and independent evolution of pathogenicity in these diverse fungi.

**Figure 3.**
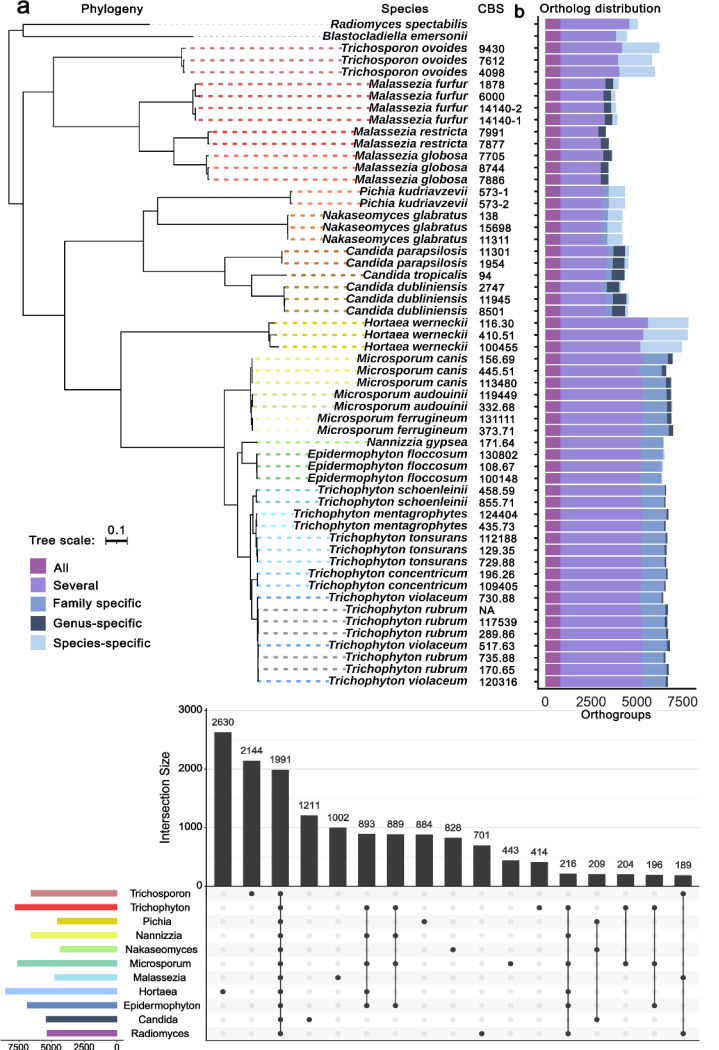
Phylogeny and distribution of orthogroups and genes in orthogroup sets. **(a)**: Phylogeny resolved by multiple sequence alignment of 827 shared genes. The two outgroups root the tree at the top. Species and CBS numbers (when applicable) as leaf IDs. Distribution of orthogroups (OG) for each sample, colored by whether the OG contains all (dark lilac), several (lilac), one family (mid blue), one genus (dark blue), or one species (light blue) of the samples. **(b)** Distribution of genera in the 17 biggest OG sets, counting OGs with at least one sample of the genus. Outgroup sample *Radiomyces* included.

We investigated the evolutionary relationships between these fungal species by constructing a maximum likelihood phylogeny based on the shared 827 OGs, with sequence alignment performed using MAFFT [17] and tree inference using FastTree [18] (Figure 3a). The inferred phylogeny was consistent with expected taxonomy. Two dermatophyte species (*T. rubrum* and *T. violaceum*) displayed non-monophyletic relationships, likely reflecting the ancient evolutionary splits represented in the tree. These deep splits occurred over short evolutionary timeframes, complicating phylogenetic inference. Even a focused analysis of pezizomycotina samples (*Epidermophyton*, *Hortaea*, *Nannizzia*, *Microsporum* and *Trichophyton*) with an outgroup still led to the inference of a non-monophyletic relationship. While *T. violaceum* is considered part of the *T. rubrum* species complex with established genomic similarities [19], these species are phenotypically distinguishable [20], highlighting the complex relationship between morphology and genomics.

Analyses of family-, genus-, and species-specific OGs revealed patterns consistent with evolutionary relationships (Figure 3a). The closely related dermatophytes showed almost exclusively family-specific OGs and very few species-specific and genus-specific OGs. Regarding the broader phylogenetic tree, we observed more species-specific OGs within *Nakaseomyces* and *Pichia* compared to *Candida*. Among Basidiomycota, *M. furfur* displayed more species-specific OGs than the more closely related *M. globosa* and *M. restricta*. The phylogenetically distant *Hortaea* and *Trichosporon* showed the highest number of species-specific OGs (Figure 3a).

At the genus level, *Candida* possessed the largest OG set with 1,211 OGs, followed by *Malassezia* with 1,002 OGs (Figure 3b). At the family level, all genera of the *Arthrodermataceae* shared 889 OGs, while *Saccharomycetaceae* genera shared 209 OGs. Together, these analyses reveal both the conservation of core gene families essential for fungal biology and the emergence of species-specific gene sets that potentially contribute to the unique adaptive capabilities of these fungi closely associated with SFIs.

### Metabolic adaptation revealed by functional genomic analysis

**Figure 4.**
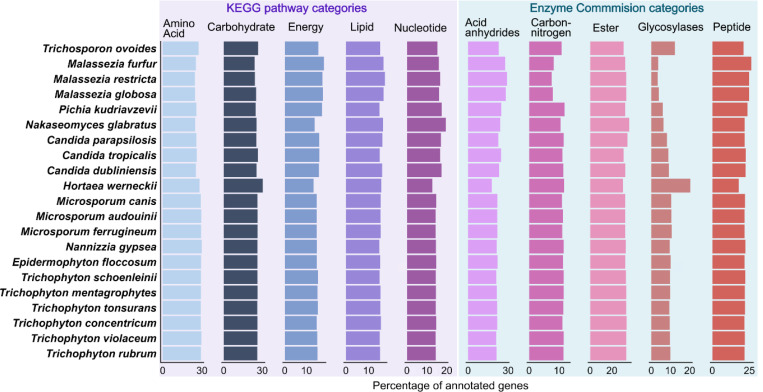
Relative functional annotations of orthogroups across species. Annotation of orthogroups with KEGG pathway metabolism categories (purple), and Enzyme Commission hydrolase (EC: 3) subcategories (blue), given as the average percentage of total annotations of that category in each species.

We next used eggNOG mapper [21] to functionally annotate 19,737 OGs using several databases including Cluster of Orthologous Genes (COG), Kyoto Encyclopedia of Genes and Genomes (KEGG), and Enzyme Commission number (EC) [22] (see Methods for details). Our analysis revealed that species within the same genus had similar functional profiles, while modest differences were found between genera. We compared metabolic pathways from KEGG and selected EC hydrolases between species (Figure 4).

*Malassezia* species showed reduced carbohydrate metabolism and slightly increased lipid metabolism compared to other genera (Figure 4). They also had fewer EC glycosylases and carbon-nitrogen targeting enzymes, aligning with previously reported gene loss in especially carbohydrate-degrading enzymes as these species became dependent on host lipids as a carbon-source [10]. In contrast, both *Hortaea* and *Trichosporon* contained higher proportions of carbohydrate metabolism genes, supported by more glycosylases and fewer peptide and acid anhydride targeting hydrolases, reflecting their plant-degrading geophilic nature. Among dermatophytes, functional annotations were remarkably similar across species, regardless of their ecological niches. The *Saccharomycetaceae* family showed variation in the hydrolase distribution, with *Nakaseomyces* and *Pichia* containing fewer glycosylases and more ester and peptide targeting enzymes, respectively. This divergent evolution between genera is not apparent in terms of clinical presentation and body-site niches. Still, these variations have potential molecular importance since they may allow the utilization of different energy sources in competitive environments.

In conclusion, our functional analysis highlights distinct metabolic profiles between taxonomic groups in this study, providing insight into different adaptations to the skin environment.

### Distribution of secreted enzymes reflects species-specific host adaptation mechanisms to the skin

**Figure 5.**
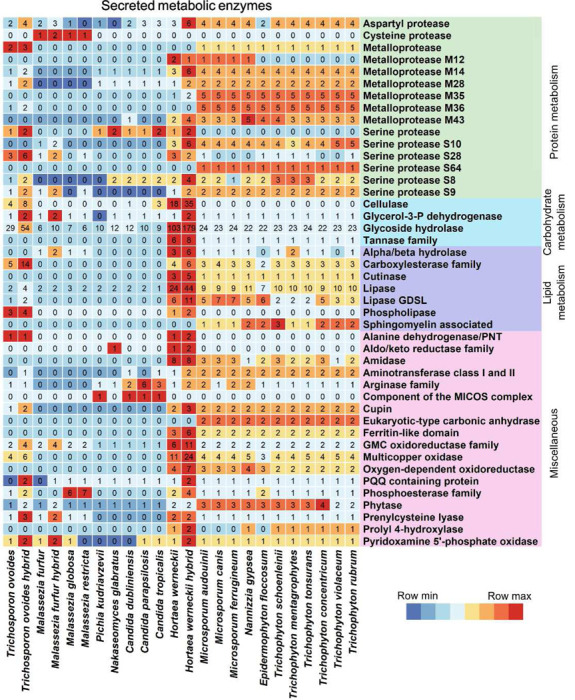
Copy numbers of secreted metabolic genes across species. Predicted secreted genes with annotated metabolic activity acting on either protein (green), carbohydrate (blue), lipid (purple) or other (pink) substrates. Colored by row z-score from minimum (blue) to maximum (red). Species gene counts summarized by rounded mean of strain counts, and hybrid strains are split from non-hybrid strains in the relevant species.

To further understand how different fungi adapt to living on human skin, we analyzed the enzymes they secrete using SignalP [23]. This analysis yielded 1,141 OGs of predicted secreted proteins, including 229 genes that still remain undescribed (supplementary table 1). We focused on genes involved in energy assimilation from the host, comparing gene copy numbers across different fungal species, with hybrid species being split into hybrid and non-hybrid strains (detail in Methods, Figure 5).

Dermatophytes showed enrichment in all types of secreted peptidases except cysteine, consistent with their keratinophilic nature where they rely on the degradation of skin and nail proteins as carbon-source. The degradation of structural proteins in the skin is relevant to the pathogenicity and virulence of keratinophilic fungi during infection and invasion as it activates the host immune system and leads to an inflammatory response [24]. In contrast, yeasts from the *Saccharomycetaceae* family*, Malassezia* and *Trichosporon* generally exhibited lower copy-numbers of secreted protein-targeting enzymes, including a genus-specific secreted (cysteine) proteinase in *Malassezia.* While these species do not rely on proteins as a carbon-source they still use peptidases to invade host tissue. We also found several potential pathogenesis-related peptidases that lacked the typical secretion signal (supplementary table 2), suggesting alternative secretion methods or limitations in N-terminal assembly.

Carbohydrates are present on the skin surface in the form of glycolipids and glycoproteins. Breakdown of external polysaccharides is central to energy assimilation for most fungi and is involved in pathogenesis and host immune activation in plant-pathogens [25]. Increased growth and infections by *Candida* spp. in patients with diabetes have been proposed to be exacerbated by the increased availability of sugars due to hyperglycemia [26].

Compared to *Malassezia* and *Saccharomycetaceae*, we observed higher numbers of secreted carbohydrate-targeting enzymes in the dermatophytes, *Hortaea*, and *Trichosporon* (Figure 5). Some of these enzymes are involved in plant matter degradation, which is expected for the two latter geophiles. The host-dependent *Trichophyton* still contains genes involved in the degradation of plant material (supplementary table 3), a testament to ongoing evolution and an illustration that gene loss can be a slow process. In contrast, the lack of such enzymes in *Malassezia* may be attributable to the extensive host-adaptation these species have undergone, involving genome compaction and a shift to lipid-dependent metabolism [27].

Lipolytic activity plays a significant role in both growth and virulence of skin fungi [28]. Lipid-dependent *Malassezia* uses external lipids as a central energy source due to their lack of a fatty acid synthase [10]. Our analysis confirmed the absence of fatty acid synthase genes in all *Malassezia* species while identifying these genes in all other examined fungi (supplementary table 4). Skin disorders associated with *Malassezia* are suggested to be induced by the breakdown of skin surface lipids into free fatty acids [29]. Interestingly, we observed fewer secreted lipases in *Malassezia* than expected (Figure 5), partly attributable to missing or fragmented signal peptides in 17 to 19 lipid-degrading enzymes (supplementary table 4).

In the dermatophytes, we identified several secreted sphingomyelin-targeting enzymes, including sphingomyelinase D, which target and hydrolyze the mammalian cell membrane lipid sphingomyelin. This breakdown is most likely implicated in virulence rather than energy assimilation and is a well-described virulence factor in spiders and bacteria [30]. In these organisms, hydrolysis of cell membranes induces cell lysis and facilitates invasion, a mechanism that could also be utilized by the skin-associated fungi. We identified non-secreted phospholipase D in all samples (supplementary table 4).

*Hortaea* was enriched in secreted lipid-targeting enzymes, consistent with the previously proposed importance of lipolysis for energy assimilation since *Hortaea* is unable to break down keratin [31]. One OG was predicted to be a phospholipase, although phospholipase activity was not detected in functional assays, which was suggested to contribute to the non-invasive lifestyle of *Hortaea* [32]. The phospholipases we identified in Hortaea might target plant sphingolipids, which would align with the non-invasiveness in animal tissue.

The variety and abundance of secreted enzymes are essential for the adaptation and pathogenesis of the fungi. These skin-associated fungi exhibit different genetic adaptations, demonstrating how different energy assimilation strategies can sustain life within the same niche, yet result in varying levels of invasiveness and distinct clinical presentations.

### Insights into infection through adhesion mechanisms

**Figure 6.**
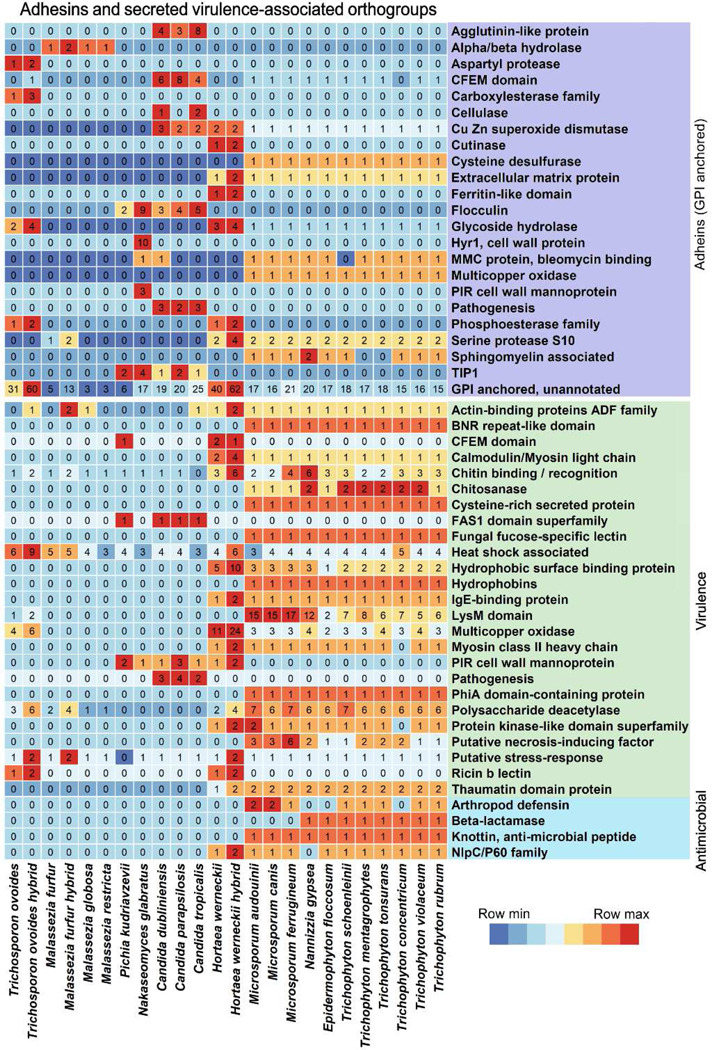
Copy numbers of secreted adhesins, virulence-associated, and antimicrobial genes across species. Predicted secreted genes with predicted GPI-anchors (purple), annotated virulence-associated function (green) or antimicrobial function (blue). Colored by row z-score from minimum (blue) to maximum (red). Species gene counts summarized by the rounded mean count within species, and hybrid strains are split from non-hybrid strains in the relevant species.

An essential factor in the pathogenicity and virulence of skin-residing fungi is their ability to adhere to host tissue and abiotic surfaces such as catheters. Adhesion proteins are also involved in flocculation, the formation of large surface-attached fungal communities [33]. Most known adhesins are large proteins anchored by covalent binding from a glycosylphosphatidylinositol (GPI) modification to membrane glucan, such that their extracellular N-terminal domain can partake in protein-ligand interactions with various host-cell surface substrates [7]. We used NetGPI 1.1 [34] to predict which of the secreted OGs had GPI-anchor sites. A total of 202 OGs containing 1,597 genes were predicted as having GPI-anchor sites (Table 3, Figure 6). The highest numbers of potential adhesins were predicted for *H. werneckii* and *T. ovoides* and fewest were identified in *Malassezia*. *N. glabrata* has the highest copy numbers relative to the number of unique OGs (when omitting the hybrids), which points to a gene expansion in some of the GPI-anchored genes in this species (Table 3).

One well-described adhesin family is the agglutinin-like sequence family, whose primary function is adherence to surfaces and which plays a key role in virulence in mice [35]. We identified agglutinin-like domains in six OGs which contain between three and eight genes in our *Candida* species, but none in *Nakaseomyces* or *Pichia* (Figure 6). Eight GPI-anchored OGs were annotated as Flocculin (FLO), with genes in all *Saccharomycetaceae.* The FLO family is diverse and involved in flocculation, biofilm formation, and the switch to filamentous growth which aids invasion and affects pathogenicity [36][37]. Hyphally regulated protein (Hyr) is another type of adhesin which is expressed during hyphal formation and is involved in resistance to phagocyte killing [38]. We found two Hyr1-annotated GPI-anchored OGs with 10 gene copies in *N. glabratus,* and observed multiple OGs annotated as Hyr1 in both *Candida* and *Nakaseomyces* without predicted GPI signals (supplementary table 5). None of the Hyr1 annotated OGs are shared between these two genera, which indicates that substantial divergence and potential gene family expansion occurred after their evolutionary split. This pattern aligns with functional divergence, as Hyr1 remains important for the pathogenicity of *N. glabratus* despite its inability to form true hyphae [39]. We also found Flocculin-annotated genes without GPI signals in all but *Trichosporon* and *Malassezia*. Previous studies have shown different cell wall attachment mechanisms for some adhesins such as those containing a C-terminal lectin-like GLEYA domain [40]. We annotated these domains in *Trichosporon* and *Saccharomycetaceae* with at most 22 gene copies in *N. glabratus*.

Common annotation terms among the rest of the predicted GPI-anchored adhesins included glycoside hydrolase, SRP1/TIP1-like Cell Wall Component and CFEM domain, while many were unannotated. Some of these could represent previously undescribed adhesins, especially in less researched non-*Saccharomycetaceae* species. Adhesins play an important role in infection and in biofilm formation, which leads to heightened virulence by antifungal resistance and increased mortality [41]. While sparse knowledge is available on biofilm formation outside the *Saccharomycetaceae,* species within dermatophytes, *Malassezia* and *Trichosporon* have been shown to form biofilms *in-vitro* [42][43][44][45]. This points to the need for further research into adhesins in a broader set of fungal species, as gaining insights into the mechanics of infection and biofilm formation can be instrumental in improving antifungal therapies and combating rising resistance.

### Analysis of genes linked to pathogenicity and virulence

Beyond adhesins, the comparative genomic analysis identified diverse virulence-associated genes among secreted OGs (Figure 6). These genes facilitate pathogenicity through invasion mechanisms, immune modulation, and the interplay with resident skin microbiota. Comparative analysis revealed that dermatophytes and *Hortaea* contain an expanded repertoire of secreted virulence-associated OGs compared to the other taxonomic groups, with few exceptions such as the heat shock associated, and PIR annotated OGs.

Dermatophytes displayed significant enrichment of the LysM domain, with copy numbers ranging from 2 in *E. floccosum* to 17 copies in *M. ferrugineum*. Previous reports established enrichment of the LysM domain in dermatophytes and proposed a link to pathogenicity and virulence through host immune evasion and keratin degradation [46][47][48]. The functional mechanism involves sequestration of chitin in the fungal cell wall, thereby preventing it from recognition as a pathogen-associated molecular pattern and subsequent degradation by host chitinases [49]. We also observed chitin-binding and chitosanase-annotated OGs with varying copy-numbers across species, potentially facilitating immune evasion and cell wall remodeling essential for tissue invasion. Additionally, we detected IgE-binding OGs in dermatophytes and *Hortaea*, suggesting potential roles in modulating the inflammatory response during the progression of infection.

Stress response mechanisms represent important virulence determinants enabling fungi to withstand host immune defenses. Our analysis identified multiple OGs associated with the stress-response, including heat shock proteins and enzymes that neutralize host-generated reactive oxygen species (Multicopper oxidase, Cu Zn superoxide dismutase) [50]. Several of these determinants were also present among the GPI anchored OGs, including CFEM domain containing genes that were notably abundant in *Candid*a. These are well-known to be involved in pathogenesis through stress-response pathways and iron-acquisition from host heme during invasion [51][52].

We also identified potential modulators of fungi-bacteria interactions within the skin microbiome. Gene families such as knottin, arthropod defensins, and NlpC/p60 have been proposed to have antibacterial effects in other organisms [53][54][55], suggesting that fungi might secrete these gene products to compete with co-resident bacteria. Collectively, this systematic characterization of virulence determinants across multiple fungal lineages establishes a molecular framework for understanding pathogenesis mechanisms.

## Discussion

In this study, we cultured, sequenced and analyzed the genomes of 51 strains across ten genera, focusing on clinically relevant fungi that cause prevalent SFIs and pose a significant health burden globally. Our comparative genetic analyses provide new insights into fungal adaptation and function, including predicting secretomes and virulence factors across diverse species.

We found high intra-genus similarity in the distribution of gene functions and notable differences between genera, including differences in predicted secreted metabolic gene products that agree with the body-site or environmental niches of the fungi. Our analyses revealed a range of adaptations to life on human skin, from host-lipid dependency in commensal *Malassezia* to an abundance of plant-degrading enzymes in free-living *H. werneckii.* We also identified adhesins, which were abundant, especially in *N. glabratus* and *Candida,* together with many previously undescribed adhesins that are potentially important for infection and biofilm formation in newly examined fungi. Finally, we compared secreted gene products involved in virulence, which revealed group-specific immune-evasion, stress-response and competition factors. Collectively, our findings reveal genomic mechanisms underlying adaptation, pathogenicity, and virulence in skin-infecting fungi.

We report two previously undescribed strains of *Trichosporon ovoides* with small genomes, suggestive of non-hybrid origins. This finding is significant as hybridization has been reported in multiple human pathogenic fungi such as *Cryptococcus, Malassezia* and *Candida,* and is likely to be an important evolutionary mechanism that contributes to the emergence of virulent traits [56][57][58]. Although hybridization may initially reduce fitness due to genetic incompatibility among two diverged lineages, overcoming these incompatibilities or experiencing hybrid vigor can enable the highly adaptable fungal genomes to undergo significant phenotypic shifts, driving pathogenicity and fitness. The coexistence of hybrid and non-hybrid lineages within the *Trichosporon* genus presents a valuable opportunity to investigate the genomic determinants of virulence through comparative approaches. By contrasting the non-hybrid strains identified in this study with known hybrid lineages, we can potentially disentangle the specific genomic features that contribute to pathogenicity.

We observed a consistent pattern of reduced genomes in the host-associated species compared to the mainly free-living species. For instance, *Malassezia* underwent gene loss and genome compaction as it developed host dependency [10]. Compared with the mainly host-associated *Candida* and dermatophytes, extensive genome compaction in *Malassezia* could indicate a more ancient host-switch event. Host switching is a well-known process in skin-associated fungi and is believed to have given rise to the highly virulent strain *T. indotineae* as a predominantly anthropophilic clonal off-shot of the zoophilic *T. mentagrophytes* [59]. This emerging pathogen, which has recently been detected beyond its endemic regions [60], highlights the role of host switching in the emergence of new, potentially drug-resistant, skin-infecting fungi.

Our comprehensive analysis of the metabolic enzyme landscape in fungal species revealed marked differences between taxonomic groups. This might reflect adaptation after a change in available energy-sources in the host, which leads to loss of genes degrading nutrients that are not present in the new niche. We discovered proportionally fewer carbohydrate-targeting enzymes and more hydrolases in *Malassezia* than other skin-associated fungi. This pattern, in combination with the absence of a fatty acid synthase gene, is likely central to the shift towards lipid-dependent commensals [27]. In dermatophytes, carbohydrate-targeting enzymes are less abundant and protein degrading enzymes are more abundant compared with closely related free living fungi [46]. The metabolic adaptations in the genome are likely to be a consequence of dermatophytes becoming dependent on host proteins as a carbon source. The secretion of metabolic enzymes is also vital for the pathogenicity of the skin-associated fungi as the breakdown of host molecules is integral to the clinical manifestations. The breakdown of lipid by *Malassezia* on the skin surface is usually asymptomatic. However, an overly high fungal load can lead to excessive release of azelaic acid, which is believed to affect the skin’s melanocytes and induce the pigmentation changes observed in pityriasis versicolor [29][61]. Examination of other genomic measures will bring more detailed insights into adaptation in skin-associated fungi, such as analyses of gene expression and evolutionary rates.

Upon invasion, adhesins play essential roles in the initial adherence to host cells by binding host cell surface structures and in the formation of biofilm by mediating cell-cell adhesion and aggregation [62]. We identified varying numbers of adhesins across skin-associated fungi, with a high proportion of undescribed proteins in all taxa except *Saccharomycetaceae.* This aligns with available research on adhesins, which has primarily focused on *C. albicans* and other *Saccharomycetaceae* [63]. Biofilm formation is clinically important, as it provides protection against the host immune system and antifungal medicine and severely complicates infections [64]. Besides *Saccharomycetaceae,* the ability to form biofilms has also been observed in *Malassezia*, dermatophytes and *Trichosporon* [42][43][44][45]. Further investigations into the implications of biofilm formation for infection could also have consequences for the treatment of conditions like chronic onychomycosis, which are prevalent and difficult to treat [65].

In addition to GPI-anchored genes, we also identified multiple adhesin annotations which were not predicted as secreted or carrying GPI-anchors. This could be an effect of the tandem repeats and genomic positions close to the telomeres, which are characteristic of adhesins [66], making complete assembly of short-read data especially challenging. Alternative surface attachment mechanisms might also be employed by the fungi, such as seen in the *N. glabratus* Epa adhesins that lack the GPI-anchor attachment signal [40]. Ultimately, we present many potential adhesin genes across the skin-associated fungi, but only few of them are well described and functionally understood. Our data will allow future assays to illuminate the function and effect on adherence of these genes, thus expanding the current understanding of adhesions and biofilm formation.

While our study provides insights into the genomics of skin-associated fungi, several limitations should be acknowledged. Short-read sequencing technology potentially results in fragmented contigs and incomplete resolution of repetitive regions and may have affected our ability to fully characterize certain genomic elements, particularly in complex regions or those containing repetitive sequences. Additionally, the functional annotation of many genes remains incomplete due to the limited reference databases for fungal species, especially those that are understudied. Many genes were assigned putative functions based solely on sequence homology, and a significant number remained annotated as hypothetical proteins with unknown functions. While the gap in functional characterization limits our understanding of species-specific adaptations and virulence mechanisms, our data should drive such work forward and provide specific avenues for future virulence studies.

It is also important to note that the presence of genes does not necessarily translate to protein expression or functional activity. As observed in morphological studies, many fungal species exhibit different morphologies based on external factors despite similar genomic content [67]. Morphogenesis, which is essential to the pathogenicity of for example *Candida* and *Malassezia* [68][69], is regulated by complex gene expression networks rather than simply gene presence or absence. This disconnect between genotype and phenotype complicates our ability to draw direct conclusions about pathogenicity or morphological characteristics from genomic data alone. Despite these challenges, our study significantly expands the genomic resources available for these understudied fungal pathogens and provides a foundation for future investigations into their biology, ecology, and pathogenicity.

In conclusion, this study demonstrates that while skin-infecting fungi employ diverse pathogenic mechanisms, their genomic adaptations provide a window into both their evolutionary history and potential clinical vulnerabilities. Our findings provide a foundation for future functional genomics and proteomics studies, which can now focus, for example, on expression pattern analysis during colonization and infection.

## Material and methods

### Fungal reference samples

Based on global reviews on the epidemiology of superficial fungal skin and nail infections published prior to 2018, we identified the most skin-relevant fungi (commensal and pathogenic, n=22) [70][71]. To investigate the inter- and intraspecies diversity of the genomes, up to four different strains of each species were obtained from the Westerdijk Fungal Biodiversity Institute biobank (Westerdijk, Utrecht, The Netherlands) and Statens Serum Institut (SSI, Copenhagen, Denmark). To obtain enough fungal biomass to perform WGS the fungi were cultured at the Department of Dermatology and Allergy, Herlev-Gentofte Hospital, Hellerup, Denmark, on appropriate media (table 1). All fungal samples were incubated at 28°C for up to 21 days, depending on their growth rate, and harvested at the stationary phase. All samples were frozen without any media and kept at −80°C until use.

### Whole genome sequencing

Colony material was transferred to 15 mL tubes including 2–3 mL lysis buffer (NucliSENS easyMag, bioMérieux Nordic, Gothenburg, Sweden) and incubated up to three days. Subsequently, all material was separated in three bead beating tubes containing 1.4-mm molecular-biology-grade zirconium beads (OPS Diagnostics, Lebanon, NJ, USA) and homogenized for 2 minutes at 30 Hz followed by centrifugation for 5 minutes at 16,000 g. Lysates (up to 3 mL) were then subjected to the automated NucliSENS easyMag platform, using 100 μL silica and with an output volume of 60 μL. Double-stranded DNA concentrations were measured using a Qubit meter and the dsDNA Quantification Assay (both Thermo Fisher Scientific, Roskilde, Denmark). DNA concentrations varied and although a starting concentration of >10 ng/μL was sought, several samples yielded lower concentrations but were successfully processed through WGS.

Libraries were constructed, and sequencing was performed using the Nextera XT Kit (Illumina, Little Chesterford, United Kingdom) and 300-cycle kits on the NextSeq 550 (Illumina) platform according to the manufacturer’s instructions. Quality control of the obtained sequencing data was conducted using Bifrost (https://github.com/ssi-dk/bifrost) to ensure adequate sequencing depth, species verification and identify contamination issues.

### Data processing

Preprocessing and quality assessment of the Illumina sequencing data were conducted using fastp (v0.23.2, https://github.com/OpenGene/fastp) with the parameter ““--trim_front1 4 --trim_tail1 2 --trim_front24 --trim_tail2 2 --cut_right -- cut_right_window_size 10 --length_required 80 --trim_poly_g”.

We performed de novo assembly using SPAdes v3.15.5 with the “--isolate” option [72], followed by polishing with Pilon [73]. Assembly stats were summarised using Quast v5.2.0 [74] and completeness was evaluated using BUSCO v5.6.1 [15] with closest available lineages. Kmer analysis was performed using Jellyfish v1.1.11 [75], Genomescope v2, and Smudgeplot [76] on the sequencing reads. On a few inconsistent assemblies we performed haplotig purging with purge_haplotig v1.1.2 [77].

Assemblies were annotated using funannotate v1.8.1 [78]. Funannotate first masks repetitive regions of the genome with RepeatMasker [79] and creates ab initio gene prediction consensus models with EVidenceModeler [80] after training Augustus v3.3 [81] using the BUSCO [15] fungi_odb10 data set and GeneMark [82]. Exon locations were inferred from protein alignments against the SwissProt [83] database with BLASTX and exonerate v2.4.0 [84]. Annotations were quality assessed using BUSCO v5.6.1 [15] with either Ascomycota or Basidiomycota lineage datasets using the “-m proteins” option.

We performed Average Nucleotides Identity (ANI) analysis using OrthoANIu [14] comparing our three *Trichosporon ovoides* (CBS7612, CBS4098 and CBS9430) assemblies in an all-vs-all approach to other different *Trichosporon* species acquired from NCBI genome [11]: *ovoides* (GCA_040368805.1), *coremiiforme* (GCA_039545355.1), *asahii* (GCA_000293215.1) and *inkin* (GCA_040365635.1).

### Orthologous gene analysis

Post-processing of results was carried out using R v4.4 and Python v3.11. Annotated genomes were analyzed using Orthofinder v2.5.5 [16], to identify shared orthologous genes and resolve the phylogeny of the species by multiple sequence alignment using MAFFT v7 [17] and maximum likelihood tree inference using FastTree v2 [18]. Two distantly related out-groups were added to the analysis as recommended by the instructions. These were chosen from well-established non-dikarya fungi divisions, *Radiomyces* from the Mucormycota division, and *Blastocladiella* from the Blastocladiomycota division. Annotated genomes were acquired from the National Center for Biotechnology Information (NCBI) genome database [11]: *Radiomyces spectabilis* (GCA_025331425.1) and *Blastocladiella emersonii* (GCA_025594325.1). OrthoANIu analysis [14] of a suspected misidentified *T. verrucosum* sample showed 99.9% ANI similarity to both tested *T. rubrum* assemblies but only 91.4% to the *T. verrucosum* reference. Based on this evidence, we reclassified the sample as *T. rubrum* and believe it must be a duplicate of one of the other *T. rubrum* strains. Orthogroup distribution in samples given in the Hierarchical Orthogroup (HOG) N0 output file was analyzed and summarized at various taxonomic levels.

### Functional annotation

The longest sequence of all resulting orthogroups (OGs) of 2 or more samples was used as the representative sequence and functionally annotated using: InterproScan v5.70–102.0 [85] with SUPERFAMILY [86], PANTHER [87], and Interpro databases [88], and EggNOG-mapper v2.1.12 searching Eggnog, EC, COG, KEGG, Pfam, and GO databases [22][89][90][91][92]. Carbohydrate-Active enZYmes (CAZYs) were annotated using dbCAN v3 [93]. Searches were conducted using HMMER [94] and DIAMOND [95]. EC-numbers, COG classifications, and CAZYmes were summarized by counting the number of OGs annotated with each category in the classification, so multiple orthologous genes in the same sample were counted only once per category. To identify secreted gene products, SignalP v6 [23] was first used to predict signal peptides then transmembrane proteins were predicted with TMHMM v2 [96] and excluded. Predicted secreted OGs were appointed to different categories pertaining to metabolism and virulence based on annotation results and manual curation. Gene counts for each OG in each sample were summarized at the species level by taking the rounded average of copy numbers. Hybrid strains were kept separate from the haploid strains of the same species. Z-scores of copy numbers were calculated and capped at −2 / +2 to improve visualization, since the hybrid strains (mainly *Hortaea werneckii*) skewed the color scale undesirably. From the predicted secreted proteins, GPI anchored proteins were predicted using NetGPI v1.1 [34].

## Supplementary Material

This is a list of supplementary files associated with this preprint. Click to download.

- suptable4.pdf
- suptable1.pdf
- table1.pdf
- suptable3.pdf
- suptable2.pdf
- table2.pdf
- table3.pdf
- suptable5.pdf
- TablesComparativeanalysisofskinfungiNCOMMS2535116.xlsx
- Table1accessionstodateNCOMMS2535116.pdf

## Figures and Tables

**Table 1.** Overview of genome statistics, and strain information. CBS numbers, assembly statistics, origin information, preferred host, culture media used, and common dermatological presentations of each strain.

**Table 2.** Orthogroup statistics. Various statistics from the Orthofinder results given for each sample.

**Table 3.** Predicted GPI-anchor containing orthogroups in samples. The number of predicted unique orthogroups with GPI-anchors and the number of genes in those orthogroups for all samples.

## Data Availability

The fungal genome assemblies generated and analyzed during this study are deposited into NCBI Genomes Database under the Bioproject accession number PRJNA1260449 (website: National Center for Biotechnology Information). All data will be made publicly available prior to publishing.
